# New Numerical Method Based on Linear Damage Evolution Law for Predicting Mechanical Properties of TiB_2_/6061Al

**DOI:** 10.3390/ma16134786

**Published:** 2023-07-03

**Authors:** Weigang Fu, Junchi Ma, Zhe Liao, Huanjie Xiong, Yaoming Fu, Bin Wang

**Affiliations:** 1 Aviation Engineering Institute, Civil Aviation Flight University of China, Guanghan 618330, China; 2 Department of Mechanical and Aerospace Engineering, Brunel University London, Uxbridge UB8 3PH, UK

**Keywords:** PRAMCs, TiB_2_ particles, numerical simulation, damage factor, linear damage evolution law, 3D RVE

## Abstract

In order to study the effect of TiB_2_ particles on the mechanical properties of TiB_2_/6061Al composites, a series of 3D TiB_2_/6061Al representative volume elements (RVEs) were established based on SEM photos. This model took into account the ductile damage of the matrix and the traction separation behavior of the interface, and the linear damage evolution law was introduced to characterize stiffness degradation in the matrix elements. Mixed boundary conditions were used in the RVE tensile experiments, and the accuracy of the predicted result was verified by the agreement of the experimental stress-strain curve. The results showed that the addition of TiB_2_ particles can effectively promote the load-bearing capacity of the composite, but elongation is reduced. When the weight fraction of TiB_2_ increased from 2.5% to 12.5%, the elastic modulus, yield strength, and tensile strength increased by 8%, 10.37%, and 11.55%, respectively, while the elongation decreased by 10%. The clustering rate of the TiB_2_ particles is also an important factor affecting the toughness of the composites. With an increase in the clustering rate of TiB_2_ particles from 20% to 80%, the load-bearing capacity of the composites did not improve, and the elongation of the composites was reduced by 8%. Moreover, the high-strain region provides a path for rapid crack propagation, and particle spacing is a crucial factor that affects the stress field.

## 1. Introduction

Particle-reinforced aluminum matrix composites (PRAMCs) have been widely used in aerospace engineering and transportation due to their characteristics of low density, high specific strength, and low cost [1,2]. PRAMCs consist of an Al matrix phase and a reinforcement phase. Commonly used reinforcement phases include SiC, ZrB_2_, and TiB_2_. Among them, TiB_2_ has been frequently introduced into various aluminum alloys as a reinforcement phase, owing to its wettability, high modulus, and high hardness [3]. It is widely accepted that the addition of a reinforcement phase can improve the strength of PRAMCs to a great extent; however, it has also been shown to reduce toughness [4]. Hence, in order to enhance the mechanical properties of PRAMCs as much as possible, the mechanism by which reinforcement particles affect the composite’s mechanical properties must first be understood. Quantifying and analyzing the characteristics of particles is a great way to study this mechanism.

Recent research has shown that the proportion of particles in composites has a significant influence on the load-bearing capacity of the composites [5,6]. Lv et al. [7] fabricated hybrid aluminum matrix composites reinforced with SiC particles and carbon fibers and showed that the addition of the reinforcement phase improved the composites’ tensile strength and elastic modulus. Yang et al. [8] fabricated ZrB_2_/6061Al nanocomposites and showed that the strength of the composite was improved by approximately 1.52 times when compared with a 6061Al alloy. Moreover, related studies show that the distribution of particles is also a crucial factor for the load-bearing capacity of the composites [9,10,11]. For example, Ma et al. [12] investigated the mechanical properties of in situ Al3Ti/A356 composites and showed that the clustering of Al3Ti particles caused a decrease in ductility in the composite. Law et al. [13] investigated the effect of particle arrangement on the mechanical response of MMCs and showed that non-clustered random and highly clustered particle arrangements resulted in the highest and lowest flow stress, respectively. Theoretical analytical methods are also used in parallel to predict the mechanical properties of the composite. For instance, Weng [14] obtained values for the interfacial stress and modulus of composites using a model in which the reinforced particles were considered to be ellipsoidal. However, this method assumes particles to be simply shaped and uniformly distributed, which does not correspond with the actual observed microstructure. The accuracy of mechanical properties predicted by the theoretical analytical method needs to be improved.

Aside from experimental and theoretical analytical methods, the microstructure-based representative volume element (RVE) has also been increasingly adopted to study double-phase composites [15,16,17]. Wang et al. [16] proposed a 2D RVE of SiC/Al and studied its mechanical behavior by introducing the discrete distribution function of reinforcement. Gad et al. [18] proposed a 2D FE model to investigate the effects of particle size and volume fraction on the deformation, damage, and failure behaviors of SiC/A356. However, 2D RVE cannot accurately reconstruct the actual microstructure, resulting in inaccurate outcomes [19]. Related work shows that 3D microstructure-based modeling has an advantage when studying complicated and heterogeneous microstructures [20,21,22,23,24]. Zhang et al. [25] proposed a 3D enhanced FE model and successfully simulated the mechanical behavior of SiCp/2009Al. Nan et al. [26] proposed a computational structural model of TiB_2_/Cu composites based on the geometric characteristics of TiB_2_ particles. The accuracy of the model was verified by comparison with the experimental stress-strain curve, and the mechanical properties of the composite were studied. However, there is still a drawback to this work: the stiffness of a constitutive element is directly removed when it meets the damage criterion without applying the damage evolution law, resulting in the stress-strain curves being underestimated when element removal occurs. To avoid this problem, Ma et al. [27] proposed a 3D RVE model that adopts the linear damage evolution law. A series of computational experiments were successfully carried out to study the influence of clustering degree and volume fraction of particles on the mechanical properties of Al3Ti/A356 composites. However, this method is unsuitable for predicting the mechanical properties of Al3Ti composites reinforced with TiB_2_ particles due to the difference in the component particles’ morphology, which will affect the field of stress. In addition, most of the aforementioned literature focused on aluminum alloys or other alloys; there is still a lack of research on composites of TiB_2_/6061Al.

To overcome the issues and limitations discussed above, the microscopic geometric characteristics of TiB_2_/6061Al were extracted from the actual SEM photos in this study. A series of 3D RVEs with different weight fractions and clustering rates were established for TiB_2_/6061Al composites. The model took into account the ductile damage of the matrix as well as the traction separation behavior of the interface, and a linear damage evolution law was introduced to characterize the stiffness degradation of the element. Furthermore, mixed boundary conditions were used for the RVE tensile experiments. The accuracy of the model was verified using experimental curves. Finally, the effects of particle weight fraction and clustering rate were studied, and the mechanical properties of TiB_2_/6061Al were predicted and assessed from three aspects: the interface damage, the load-bearing capacity of particles, and the matrix damage.

## 2. Finite Element Model

### 2.1. Material Constitutive and Fracture Behavior

A stable and reliable FE model is useful for obtaining accurately predicted results, for which it is particularly important to obtain accurate material parameters. Based on previous works, the mechanical properties of TiB_2_ particles and the 6061Al matrix are presented in Table 1. The plastic hardening behavior of 6061Al is described by the exponential formula expressed as [28]:

(1) $$\sigma =\sigma_{y}{\left(1+\frac{E}{\sigma_{y}}\varepsilon_{p}\right)}^{n}$$

where *σ* is stress, *E* is the elastic modulus, and *σ_y_* and *ε_p_* are the yield strength and plastic strain, respectively. The exponent *n* is 0.17, which was obtained by fitting a uniaxial tensile stress-strain curve for the 6061Al alloy.

The fracture behavior of TiB_2_ particles is normally not taken into account by FE models due to the fact that the fracture of particles is not observed in tensile tests of the composites [29,30]. Hence, only the linear elastic behavior of TiB_2_ particles was considered in this paper. Ductile damage is the main cause of failure in the matrix of Al alloys. Based on the Rice and Tracey criterion [31], the damage factor *D* was used to characterize the degree of ductile damage in the Al matrix:

(2) $$D=\int_{0}^{{\bar{\varepsilon }}^{p}{}^{l}}e^{\frac{3}{2}\eta }d{\bar{\varepsilon }}^{pl}$$

(3) $${\bar{\varepsilon }}^{pl}=\int_{0}^{t}{\dot{\bar{\varepsilon }}}^{pl}dt,{\dot{\bar{\varepsilon }}}^{pl}=\sqrt{\frac{2}{3}{\dot{\varepsilon }}^{pl}:{\dot{\varepsilon }}^{pl}}$$

where $\eta =\sigma_{m}/\sigma_{y}$ is stress triaxiality, $\sigma_{m}$ is the hydrostatic pressure, $\sigma_{y}$ is the total stress, ${\bar{\varepsilon }}^{pl}$ is an equivalent plastic strain (PEEQ), and ${\dot{\varepsilon }}^{pl}$ is the plastic strain rate tensor.

**Figure 1:**
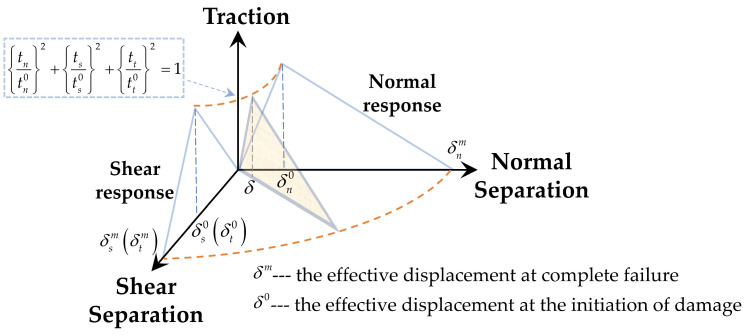
Bilinear cohesive model.

During the experimental process, the damage factor *D* accumulates continuously in each element with the increase in strain. When *D* reaches a specified value, failure of the matrix elements occurs. It should be noted that the post-failure behavior of the matrix plays a significant role in predicting the stress-strain curves of the failure stage. However, in previous work on composites [32], the element that retained stiffness was immediately deleted when it satisfied the failure criterion during the failure stage. Although this method can simulate the process of crack propagation, the predicted stress-strain curve is underestimated compared to the experimental result. In this study, a linear damage evolution criterion was used to characterize the post-failure behavior of composites [23]. The expression of this linear damage evolution criterion [33] is:

(4) $$d=\min\left(1,k_{d}\frac{\varepsilon_{eq}-\varepsilon_{0}}{\varepsilon_{0}}\right)$$

where *ε*_0_ is the initial equivalent failure strain, *ε_eq_* is the current equivalent strain, and *k_d_* is a parameter controlling the degradation rate of the material’s stiffness. When *d* reaches 1, the element’s stiffness becomes 0, and it is deleted. In order to characterize the interfacial failure in the microscale RVE model, a bilinear cohesive model (as shown in Figure 1) was introduced at the interface between the Al matrix and TiB_2_ particles to characterize its debonding behavior. A cohesive element of zero thickness was used to discretize the interfacial layer. The linear elastic behavior of the interface layer is defined by the traction separation criterion:

(5) $$t=\left\{\begin{array}{c} t_{n}\\ t_{s}\\ t_{t} \end{array}\right\}=\left\{\begin{array}{ccc} K_{nn} & 0 & 0\\ 0 & K_{ss} & 0\\ 0 & 0 & K_{tt} \end{array}\right\}\left\{\begin{array}{c} \delta_{n}\\ \delta_{s}\\ \delta_{t} \end{array}\right\}=K\delta$$

where *t* is the nominal stress vector. *t_n_*, *t_s_*, and *t_t_* are the normal and the two shear tractions, respectively, and the corresponding separations are denoted by $\delta_{n}$, $\delta_{s}$ and $\delta_{t}$. *K* is the stiffness coefficient of the interface and δ is the nominal strain of the interface.

The QUADS stress criterion was applied to characterize the damage evolution behavior of the cohesive elements. When the stress on the interface satisfies the QUADS stress criterion, the failure of a cohesive element in the interface layer occurs, and this criterion is expressed as:

(6) $${\left\{\frac{t_{n}}{t_{n}^{0}}\right\}}^{2}+{\left\{\frac{t_{s}}{t_{s}^{0}}\right\}}^{2}+{\left\{\frac{t_{t}}{t_{t}^{0}}\right\}}^{2}=1$$

where $t_{n}^{0}$, $t_{s}^{0}$, $t_{t}^{0}$ are maximum tractions in the normal and two shear directions, respectively.

However, it is difficult to obtain the material parameters of the cohesive elements of the interface layer experimentally. Generally, due to the limitations of the present experiments [34], the most reasonable parameters of cohesive elements are obtained by comparing the simulated stress-strain curve with the experimental curve [19,26,29,35,36,37,38]. By comparing the experimental curve, the parameters of the cohesive element were fixed as follows: 700 MPa for the interfacial strength and 0.05 um for the initial separation displacement. As ABAQUS does not provide the required constitutive model, a Vumat subroutine was developed for the material.

### 2.2. Quantitative Characterization of Damage Behavior

During the process of failure, the damage behavior of the matrix and interfacial layer is different. In order to quantitatively characterize the damage degree of the interface and matrix, the interface damage ratio *D_interface_* and matrix damage ratio *D_matrix_* were introduced. The corresponding expressions are as follows:

(7) $$D_{interface}=\frac{\sum_{i=1}^{N_{d}}A_{i}}{\sum_{i=1}^{N_{t}}A_{i}}\times 100\%$$

(8) $$D_{matrix}=\frac{\sum_{i=1}^{N_{d}}V_{i}}{\sum_{i=1}^{N_{t}}V_{i}}\times 100\%$$

where *N_d_* and *N_t_* represent the number of failed elements and the total number of elements, respectively. The *A_i_* and *V_i_* are status values for elements of the interface layer and the matrix.

### 2.3. Establish the RVE Model

**Figure 2:**
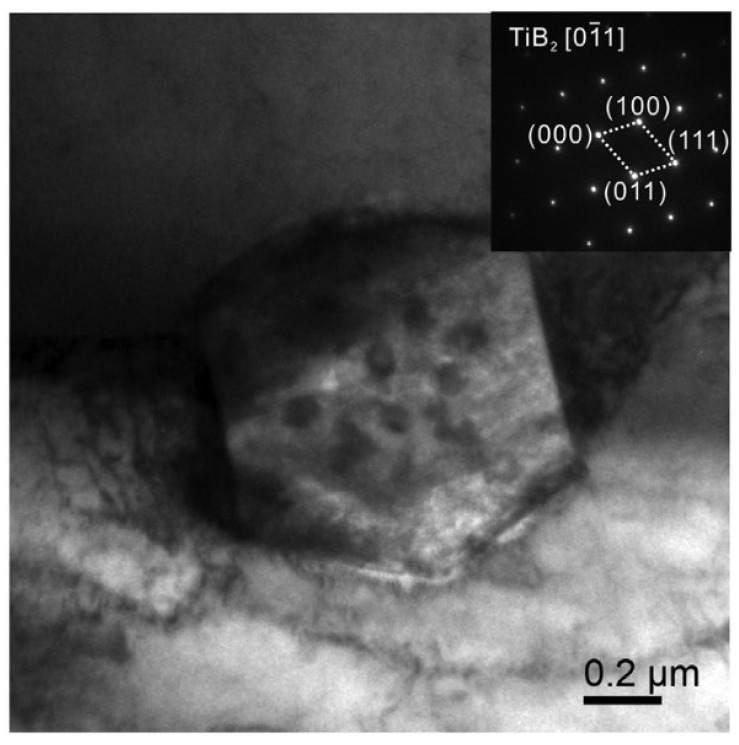
The bright-field TEM image of TiB_2_ [5].

Figure 2 is the bright-field TEM image of TiB_2_. It shows that TiB_2_ is a hexagonal prism particle with a diameter of approximately 800 nm. 

Based on the random adsorption algorithm (RSA) [39], a three-dimensional RVE model for TiB_2_/6061Al composites was established. To be adsorbed into the RVE, the location of each micro-particle is required to meet two conditions: (1) no overlapping between two particles; and (2) the distances between particles, as well as the distances from particles to the RVE faces, should exceed a certain threshold to ensure the quality of the finite elements during meshing.

The control equations of RSA can be written as follows:

(9) $$\left\|x^{i}-x^{i}\right\|\geq r_{i}+r_{j}+d_{1}$$

(10) $$\left|x_{k}^{i}-r_{i}\right|\geq d_{2}$$

(11) $$\left|x_{k}^{i}+r_{i}-L\right|\geq d_{2}$$

where *x*, *r*, and *L* represent the coordinate vector of the particle center, the particle radius, and the side length of the RVE, respectively. *d*_1_ and *d*_2_ are user-selected distances.

**Figure 3:**
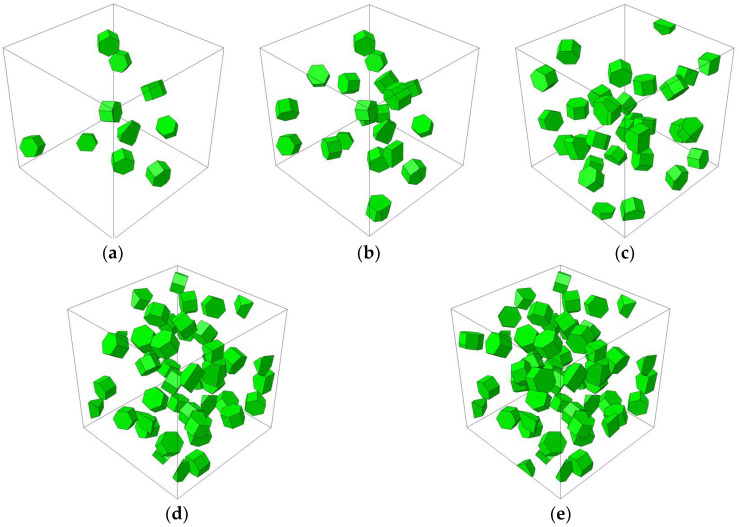
RVE models with different particle weight fractions. (**a**) *wt* = 2.5%. (**b**) *wt* = 5%. (**c**) *wt* = 7.5%. (**d**) *wt* = 10%. (**e**) *wt* = 12.5%.

**Figure 4:**
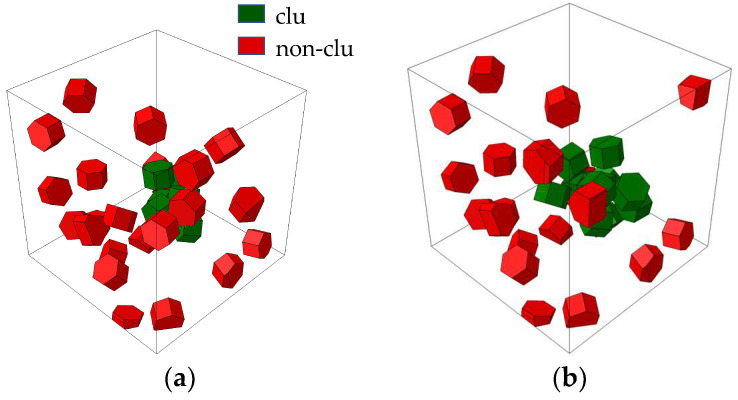

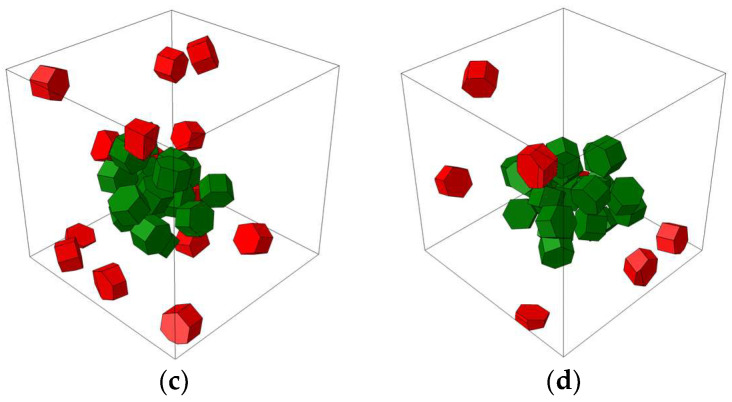
RVE models with different clustering rates. (**a**) *β* = 20%. (**b**) *β* = 40%. (**c**) *β* = 60%. (**d**) *β* = 80%.

In order to analyze the influence of microstructural parameters on the mechanical properties of composites, the TiB_2_ particle was assumed to be a hexagonal particle with a diameter of 800 nm, and the 6061Al matrix was assumed to be a continuous homogeneous material. Based on these assumptions, the RVE models were established with different particle weight fractions (*wt*) ranging from 5% to 12.5%, and different clustering rates (*β*) ranging from 20% to 80%, as shown in Figure 3 and Figure 4 (the dark green part represents clustered particles, and the dark red part represents non-clustered particles). In order to quantitatively characterize the degree of particle clustering, based on the reconstructing algorithm, several box-like subdomains were randomly generated in the cubic RVE, and a *β* parameter was introduced [40]. Its expression is as follows:

(12) $$\beta =\frac{V_{p}^{c}}{V_{p}^{t}}=\frac{\xi \sum_{i=1}^{n}V_{i}^{sub}}{\zeta V}$$

where $V_{p}^{c}$ and $V_{p}^{t}$ are the volume of clustered particles and total particles, respectively. *ξ* and *ζ* are the particle volume fraction in the subdomains and the nominal particle volume fraction in the whole RVE, respectively. $V_{i}^{sub}$ is the volume of each subdomain. *V* is the total volume of RVE.

**Figure 5:**
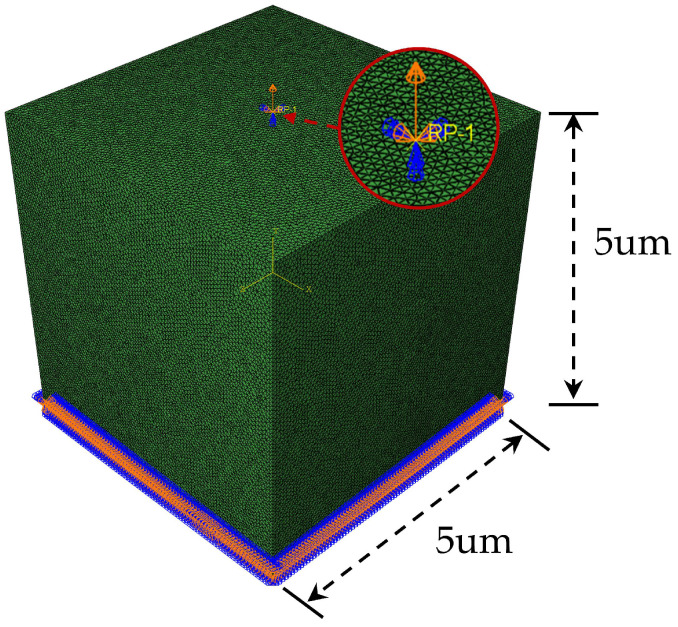
Boundary conditions of RVE.

The boundary conditions of the RVE can be divided into two types: periodic boundary conditions (PBCs) and mixed boundary conditions. Although some previous works [41] adopted periodic boundary conditions and obtained relatively accurate results, a study [19] shows that PBCs are only applicable to static and implicit numerical simulations. When they are used in explicit dynamic simulations, not only is the calculation cost high, but high-frequency oscillations occur in the model, affecting the accuracy of the numerical solution. Considering the above disadvantages of PBCs, mixed boundary conditions were adopted in this work. As shown in Figure 5, based on the previous simulation works [26,27,35,42], the RVE with a size of 5 um is established. All degrees of freedom on the bottom surface of the RVE were constrained, and the coupling command was used to couple all degrees of freedom on the top surface to the reference point. The linear displacement load was applied to the reference point, the load amplitude curve was defined by the Tabular command, and the total time of the analysis step was set to 0.01 s.

**Figure 6:**
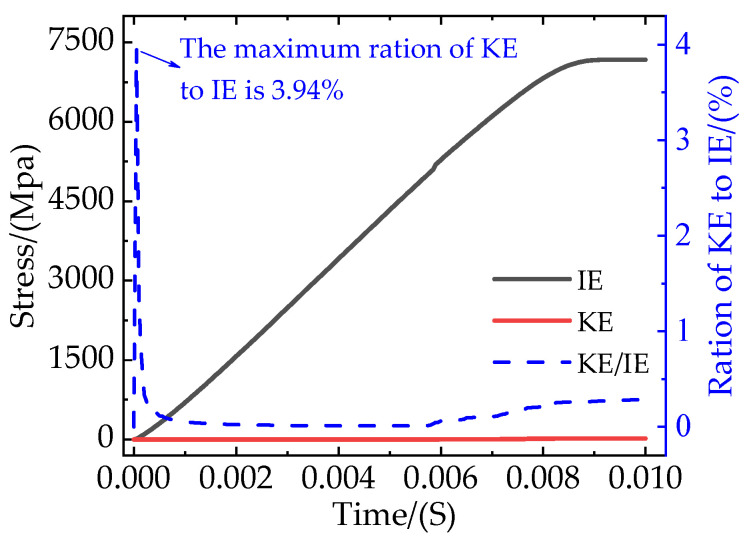
The ratio of kinetic energy to internal energy.

In this paper, the ABAQUS/Explicit Dynamic Method was used to investigate the quasi-static tensile process of composites. The stability of the model is an important factor affecting the accuracy of the simulation results, and the above settings can ensure that the ratio of kinetic energy (KE) to internal energy (IE) of the model is less than 5% and that it is stable, as shown in Figure 6.

### 2.4. Model Verification

**Figure 7:**
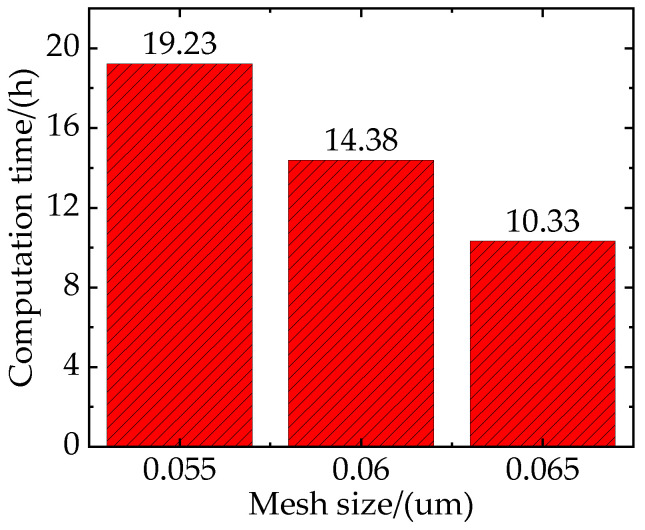
The computation time for RVE simulations with different mesh sizes.

To ensure the computational accuracy and efficiency of the RVE model, the effect of mesh size should be excluded before the simulation begins. However, as the mesh size decreases and the number of elements increases, the scale of the calculation also increases. Therefore, it is necessary to consider the balance between computational accuracy and the time taken when choosing the mesh size. As shown in Figure 7, the simulation times corresponding to the mesh sizes of 0.55 um, 0.6 um, and 0.65 um are 19.23 h, 14.38 h, and 10.33 h, respectively.

**Figure 8:**
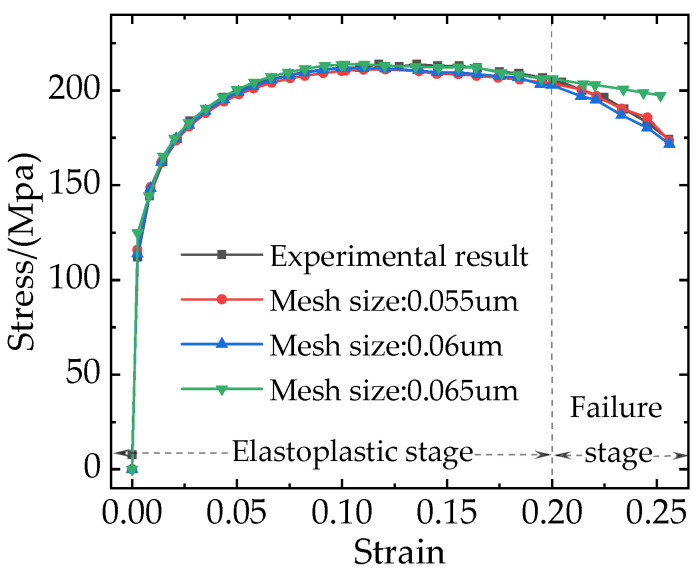
The results of RVE simulations with different mesh sizes.

The stress-strain curves of the RVE model at different mesh sizes are shown in Figure 8. It can be seen that, when the composite is in the elastoplastic stage, the effect of different mesh sizes on the stress-strain curves is not obvious. When the composite enters the failure stage, there are still no obvious differences between the stress-strain curves with the mesh sizes of 0.055 um and 0.06 um, and the FE results agree well with the experimental results [3]. However, the stress-strain curve with a mesh size of 0.065 um shows a large error. Hence, the mesh size of 0.06 um was chosen for the subsequent simulation analysis based on the balance between computing accuracy and time cost.

## 3. Results and Discussion

### 3.1. Effects of Different Particle Weight Fractions on TiB_2_/6061Al Composites

**Figure 9:**
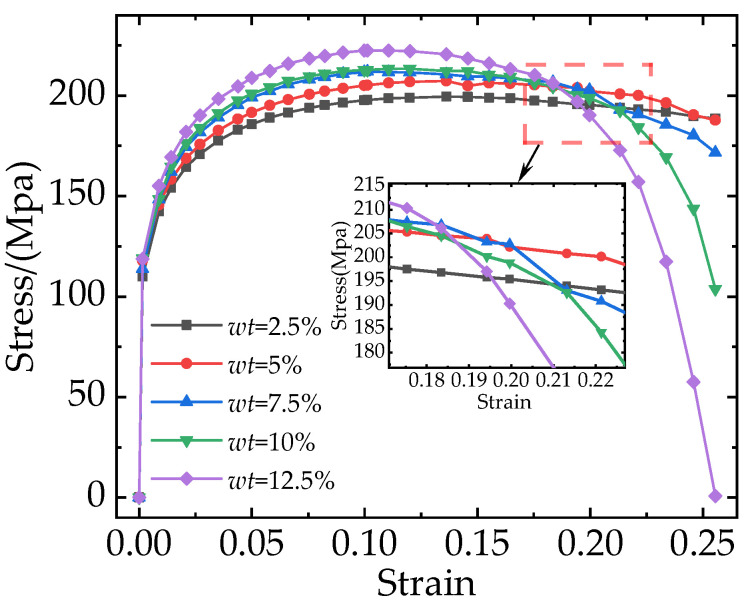
Stress-strain curves for composites with different particle weight fractions (*wt*).

For PRAMCs, the fraction and size of reinforcement particles in composites are considered crucial factors that affect the mechanical properties of composites. However, several published papers [18,27,29,36] showed that the refinement of particles would improve the strength of composites. Therefore, in this paper, we only investigate the effect of particle weight fraction on mechanical properties. Figure 9 shows the stress-strain curves of composites with different particle weight fractions. Although all the stress-strain curves initially increase and then decrease, there is a clear difference between the stress values of curves with different particle weight fractions. The composite with a higher particle weight fraction enters the failure stage earlier, and damage occurs earlier. With the increase in particle weight fraction from 2.5% to 12.5%, the elastic modulus, yield strength, and tensile strength of the composites increased from 73.33 GPa to 79.19 GPa, 164 MPa to 181 MPa, and 199 MPa to 222 MPa, respectively, whereas the elongation decreased from 25% to 15%. The experimental results show that the increase in particle weight fraction improved the load-bearing capacity of the composites.

**Figure 10:**
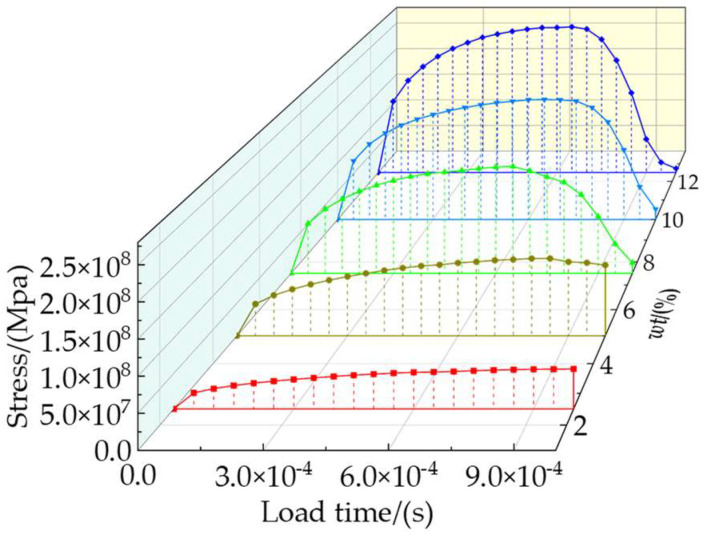
Total stress on reinforcement particles with different weight fractions.

**Figure 11:**
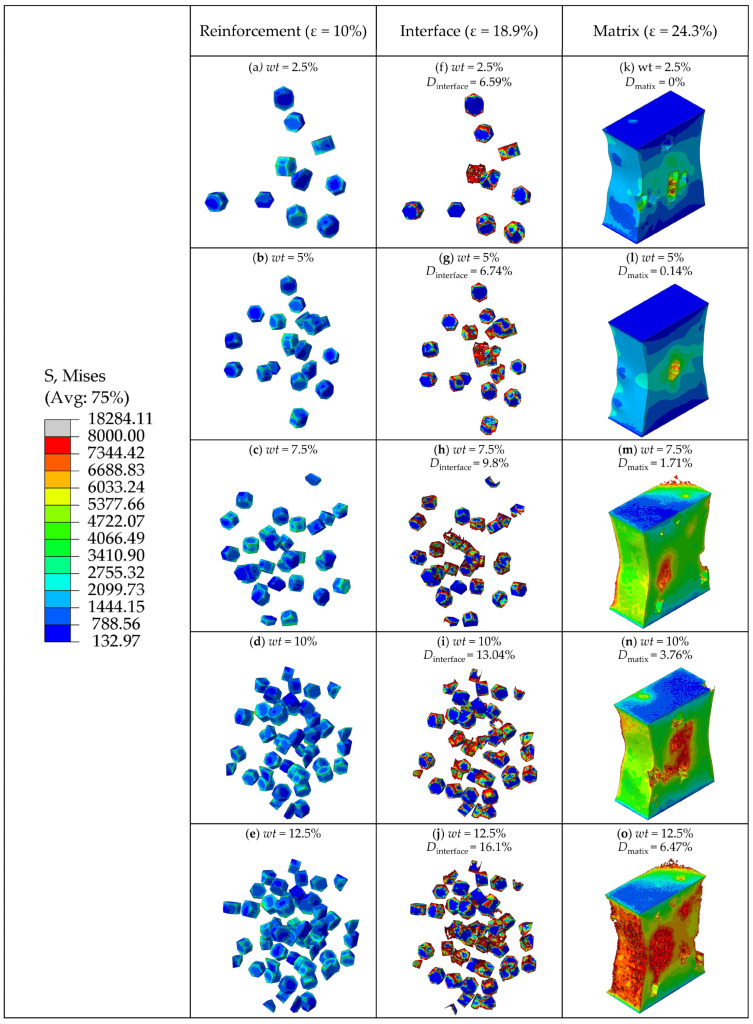
(**a**–**e**) The von Mises stress of TiB_2_ particles at the strain of *ε* = 10%. (**f**–**j**) The SDEG of the damage of the interfacial layer at the strain of *ε* = 18.9%. (**k**–**o**) The PEEQ of the matrix phase at the strain of *ε* = 24.3%.

The total stresses on the particles at different weight fractions are shown in Figure 10. And different colors in the curves correspond to different weight fractions of particles in the RVE model. The von Mises stress of the TiB_2_ particles, the SDEG of the damage to the interfacial layer, and the equivalent plastic strain (PEEQ) of the matrix phase are shown in Figure 11.

As shown in Figure 11a–e, when the strain was 10%, the number of particles loaded with high stress (more than 5377 MPa) increased. This indicates that the particles with a higher weight fraction can better bear the stress from the matrix. When the plastic deformation of composites occurs, a geometric mismatch caused by the varying degrees of strain on different parts of the composites also occurs, which causes the particles to move in different directions. It changes the original particle spacing, resulting in higher stress on neighboring particles that are close to each other and lower stress on particles that are further apart. This means that particle spacing is an important factor affecting the stress distribution. At the same time, most of the stress is concentrated on the edges and corners of the hexagonal prism particles due to the geometric discontinuity. Figure 10 shows that the total stresses borne by the particle phase increase with the increase in the particle weight fraction, which means that a higher particle weight fraction transfers stress from the matrix to the particle. Therefore, composites with higher particle weight fractions can bear higher loads.

As shown in Figure 11f–j, when the strain was 18.9% and the particle weight fraction increased from 2.5% to 12.5%, the interfacial damage ratios of TiB_2_/6061 Al composites were 6.59%, 6.74%, 9.8%, 13.04%, and 16.1%, respectively. This indicates that a higher particle weight fraction leads to more severe interfacial damage. Because most of the stress is concentrated on the edges and corners of the particles, the interfacial layers in these areas are prone to damage. Hence, it can be concluded that excessive stress is an important causal factor in interfacial failures.

As shown in Figure 11k–o, when the strain was 24.3% and the particle weight fraction increased from 2.5% to 12.5%, the damage ratios of the matrix were 0%, 0.14%, 1.71%, 3.76%, and 6.47%, respectively. Due to the increase in the number of particles, the continuity of the particles leads to a further increase in the stress on the particles [36] and the region between neighboring particles. This causes the composite with a higher particle weight fraction to become damaged earlier and more severely. Combined with Figure 11a–e, this shows that the largest stress is concentrated on the particles’ edges and corners, which eventually leads to the occurrence of matrix damage.

**Figure 12:**
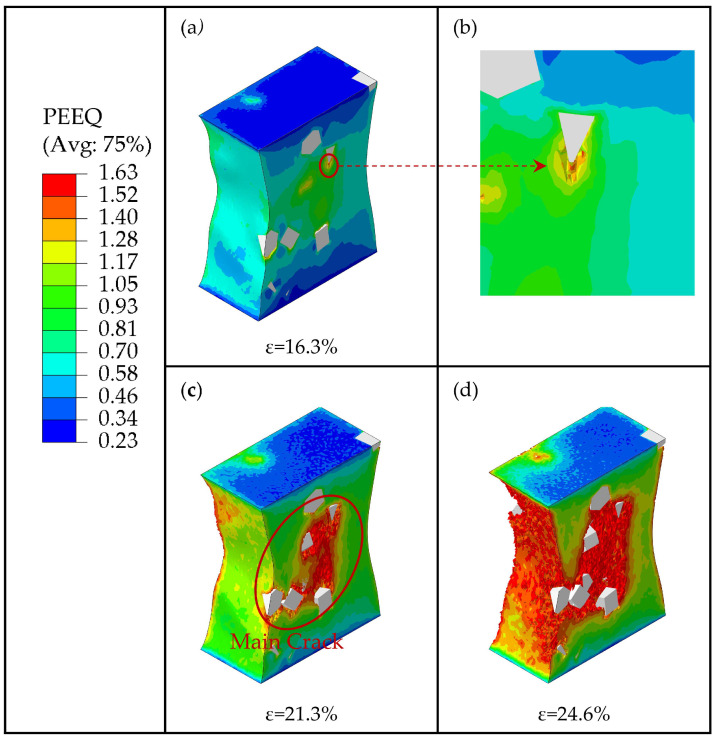
The process of crack propagation in the matrix with the particles weight fraction *wt* = 7.5%. (**a**) PEEQ of matrix at the strain of *ε* = 16.3%. (**b**) The partial enlarged view. (**c**) PEEQ of matrix at the strain of *ε* = 21.3%. (**d**) PEEQ of matrix at the strain of *ε* = 24.6%.

It is necessary to study the mechanism of crack generation and propagation because it strongly affects the elongation of composites. Figure 12 shows the PEEQ distribution contour of the composite matrix with particle weight fractions of 7.5% at strains of 16.3%, 21.3%, and 24.6%. When the strain was lower than 16.3%, no cracks were generated. However, when the strain reached 16.3%, excessive stress became concentrated on the particle edges and corners, creating high strain in these areas. This can lead to interfacial debonding and matrix damage in the high-strain area, which can further evolve into fine cracks inside the matrix. At the same time, there is also severe stress concentration at the crack tip, which causes crack propagation during the loading process. Additionally, with the increase in strain, the fine crack shown in Figure 12a,b propagated further along the edges of particles; as the strain reached 21.3%, the fine crack continued to propagate along the high-strain area and merged with other fine cracks caused by the other particles, forming a main crack (as shown in Figure 12c). Furthermore, when the strain reached 24.6%, the main crack continued to propagate and merged with the fine cracks around the other particles, forming a larger crack damage area (as shown in Figure 12d), which eventually led to the macro-level weakening of the composite’s load-bearing capacity.

### 3.2. Effect of Different Clustering Rates on TiB_2_/6061Al Composites

**Figure 13:**
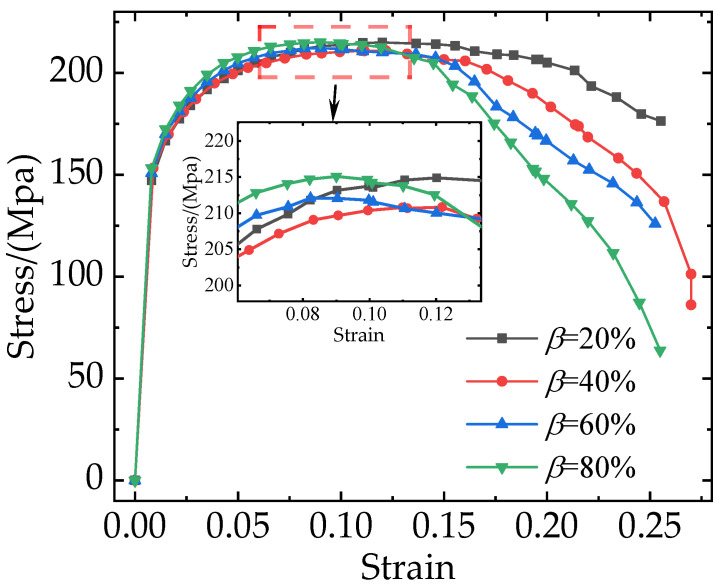
Stress-strain curves with different clustering rates (*β*).

According to previous works [27,43,44], the damage to composites is related to not only the particle weight fraction but also the degree of particle clustering. As shown in Figure 9, the RVE with a particle weight fraction of 7.5% has the most balanced mechanical properties. This section takes composites with a weight fraction of 7.5% as an example to investigate the effect of particle clustering rates on mechanical properties. Figure 13 shows the stress-strain curves corresponding to different particle clustering rates. As the particle clustering rates increase, the damage occurs earlier, and the stress on the composites decreases more rapidly during the failure stage. With the increase in particle clustering rates, the elastic modulus, yield strength, and tensile strength of the composites do not show any obvious difference. However, the elongation of the composites decreases from 22% to 14%.

**Figure 14:**
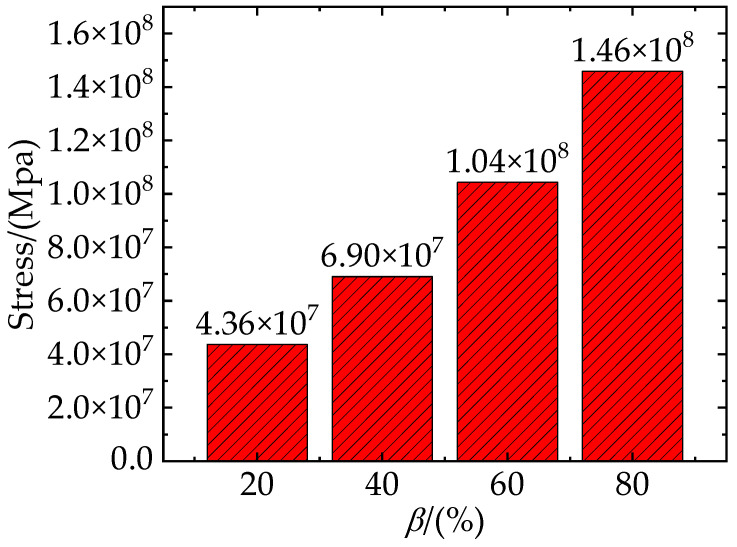
Total stress of clustered particles at the strain of *ε* = 8.1%.

**Figure 15:**
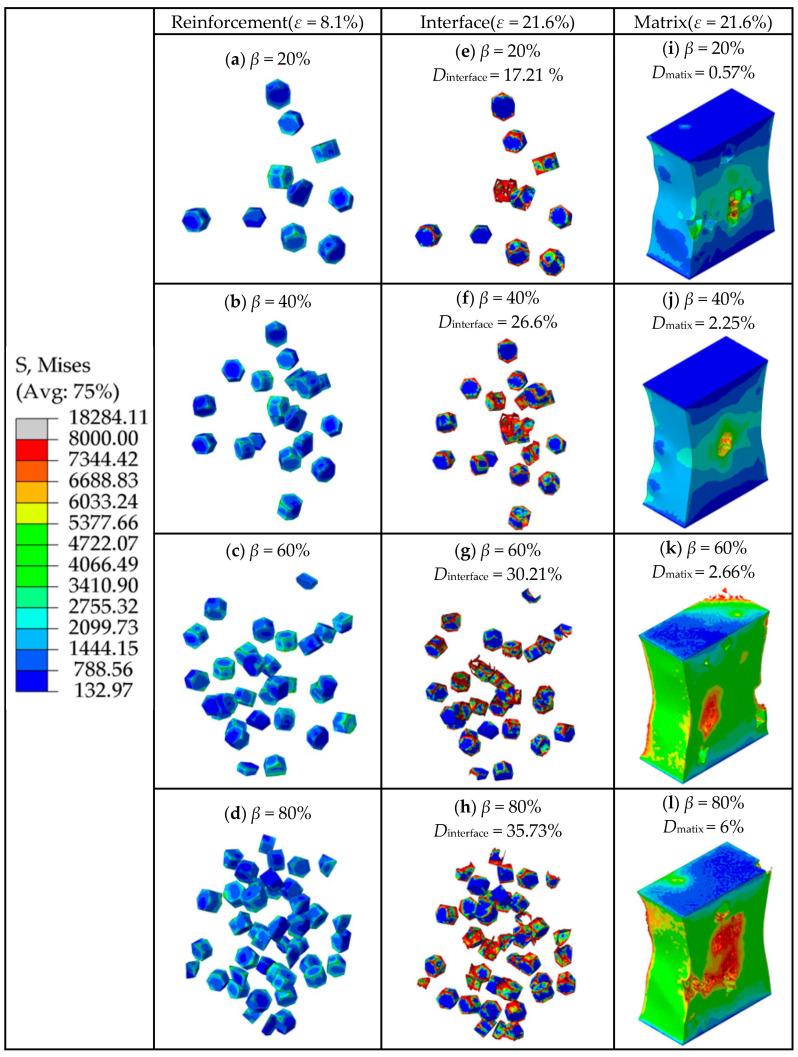
(**a**–**d**) The von Mises stress on TiB_2_ particles at the strain of *ε* = 8.1%. (**e**–**h**) The SDEG of the damage of the interfacial layer at the strain of *ε* = 21.6%. (**i**–**l**) The PEEQ of the matrix phase at the strain of *ε* = 21.6%.

Figure 14 shows the total stresses on the clustered particles with different clustering rates at the strain of 8.1%. It shows an approximately linear increase in stress in terms of *β*. Figure 15 shows the von Mises stress of TiB_2_ particles at the strain of 8.1%, the SDEG of the interfacial layer damage at the strain of 21.6%, and the PEEQ of the matrix phase at the strain of 21.6%.

As shown in Figure 15a–d, with the increase in particle clustering rate, the number of clustered particles increases. Most of the stress on the particles within the clustered particle area is between 3383 MPa and 5362 MPa, while most of the stress on the particles within the non-clustered particle area is below 3383 MPa, which indicates that the clustered particles bear a greater amount of stress than the non-clustered particles. Due to the geometric discontinuity, most of the stress is concentrated on the edges and corners of the TiB_2_ particles. Along with Figure 14, this shows that the stress borne by clustered particles increases with the clustering rate. This implies that a higher clustering rate of particles will result in a greater amount of stress borne by clustered particles.

As shown in Figure 15e–h, when the strain was 21.6%, the interfacial damage ratios of the composites were 17.21%, 26.6%, 30.21%, and 35.73%, respectively, and the clustering rate was 20%, 40%, 60%, and 80%. With the increase in particle clustering rates, the interfacial damage ratios increase, which indicates that high particle clustering rates lead to severe interfacial damage. Similar to the conclusion of Section 3.1, the damage to the edges and corners in the interface layer is more severe than in other parts. As discussed above, the clustered particles bear a greater amount of stress than the non-clustered particles. The degree of damage to the interface layer in the clustered particle area is therefore higher than that in the non-clustered particle area. By comparing the damaged area of the interface layer with the stress distribution area of the particle in Figure 15a–d, it can be seen that the damaged area of the interfacial layer is basically consistent with the area of high stress on the clustered particles, which indicates that the clustered particles are more prone to bearing high stress that exceeds the strength limit of the interfacial layer under the influence of the clustering effect, eventually leading to the failure of the interfacial layer elements.

As shown in Figure 15i–l, the matrix damage ratios gradually increase as the particle clustering rates increase, which indicates that the increase in particle clustering rates can increase the damage ratios of the matrix. Considering that the RVE model is stretched in the vertical direction, when the matrix is loaded, inward contraction strain in the horizontal direction is simultaneously generated. This contraction strain causes the particle spacing in the clustered particle area to decrease, and the stress on the particles to further increase due to the clustering effect. This causes the stress on the edges and corners of the particles in the clustered area as well as the region between the neighboring particles to exceed the strength limit of the matrix, which eventually leads to the occurrence of matrix damage.

**Figure 16:**
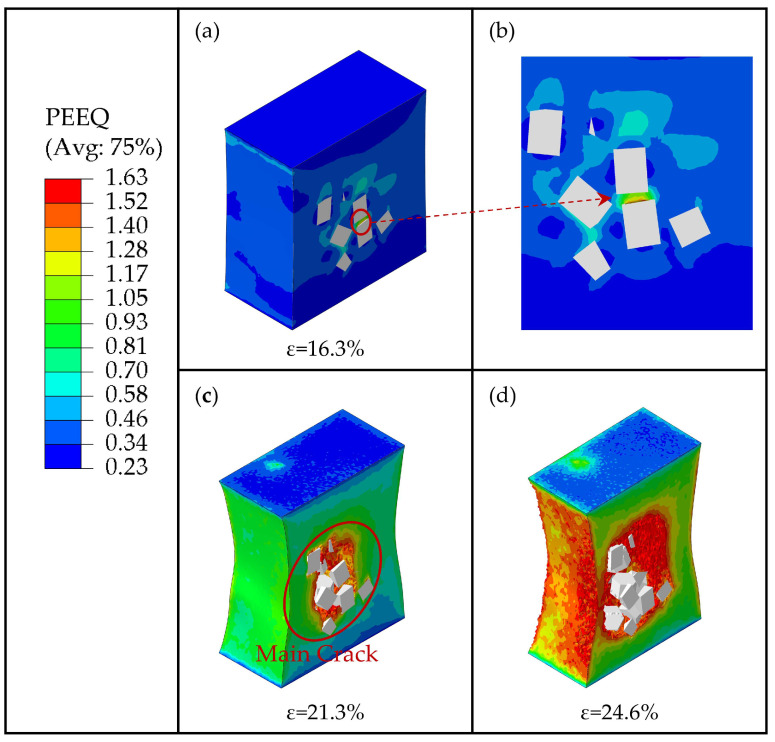
The process of crack propagation in the matrix with the clustering rate *β* = 80%. (**a**) PEEQ of matrix at the strain of ε = 16.3%. (**b**) The partial enlarged view. (**c**) PEEQ of matrix at the strain of ε = 21.3%. (**d**) PEEQ of matrix at the strain of ε = 24.6%.

As Figure 16 shows, when the strain reached 16.3%, the excessive local stress in the clustered particle area caused matrix damage to occur. As discussed above, due to the clustered particles bearing large stresses, the region between neighboring particles turns into a high-strain region. It results in the generation of an initial fine crack in the matrix (as shown in Figure 16a,b). When the strain reached 21.3%, the crack propagated further along the high-strain area and merged with other particle-induced fine cracks, eventually forming the main crack (as shown in Figure 16c). Furthermore, when the strain reached 24.6%, the main crack continued to propagate rapidly in the high-strain area and merge with the fine cracks around the other particles outside the high-strain area, forming a larger crack damage area (as shown in Figure 16d), which leads to the macro-level weakening of the composite’s bearing capacity. We conclude that the high-strain area between the clustered particles provides a fast expansion path for crack propagation.

## 4. Conclusions

In this paper, a 3D RVE model based on the actual microstructure acquired by SEM was developed for TiB_2_/6061Al composites. The mechanical behavior was predicted, and the process of fracture was simulated. By comparing the experimental results of previous studies, the accuracy of the RVE was verified. The effect of microstructural parameters of composites on the mechanical properties of PRAMC was discussed. Some conclusions are summarized below:

(1). The RVE model comprehensively considers the ductile fracture of the matrix and the traction separation behavior of the interface. It also introduces the linear damage evolution law to characterize the stiffness degradation of the element. It was successfully applied to predict the mechanical properties of composites.
(2). For TiB_2_/6061Al composites, increasing the particle weight fraction improves strength but reduces toughness. With an increase in the weight fraction of TiB_2_ from 2.5% to 12.5%, the elastic modulus, yield strength, and tensile strength increased by 8%, 10.37%, and 11.55%, respectively, whereas elongation decreased by 10%.
(3). Clustered particles also have an important effect on the internal stress field of composites. The results show that the clustering rate of particles has a great effect on toughness. With an increase in the clustering rate from 20% to 80%, the rate of decrease in stress increases constantly, and the elongation of composites decreases by 8%. However, it has little effect on yield strength, tensile strength, or elastic modulus.
(4). The mechanism of crack generation and propagation was also studied. A fine crack is first generated around the particles. Then, this fine crack merges with other fine cracks nearby, forming a main crack and eventually leading to the fracture of the composites.
(5). The high-strain region in the matrix provides a fast expansion path for crack propagation. The reduction in particle spacing causes the stress on the particles to increase and the damage to occur earlier.

## Figures and Tables

**Table 1:** Properties of components in TiB_2_/6061Al composites.

Properties	Symbols	Unit	6061Al [3]	TiB_2_ [25]
Elastic modulus	*E*	GPa	69	540
Density	*ρ*	g/cm^3^	2.7	4.52
Poisson’s ratio	*μ*	-	0.33	0.11
Yield strength	*σ_y_*	MPa	94	-

## Data Availability

The simulation results in this study are available from the corresponding author upon reasonable request.
